# Intensity and lag-time of non-pharmaceutical interventions on COVID-19 dynamics in German hospitals

**DOI:** 10.3389/fpubh.2023.1087580

**Published:** 2023-03-06

**Authors:** Yvette Montcho, Paul Klingler, Bruno Enagnon Lokonon, Chénangnon Frédéric Tovissodé, Romain Glèlè Kakaï, Martin Wolkewitz

**Affiliations:** ^1^Laboratoire de Biomathématiques et d'Estimations Forestières, Université d'Abomey-Calavi, Cotonou, Benin; ^2^Institute of Medical Biometry and Statistics, Faculty of Medicine and Medical Center, University of Freiburg, Freiburg, Germany

**Keywords:** lag-time effects, non-pharmaceutical interventions, distributed lag linear model, COVID-19 dynamics, Germany

## Abstract

**Introduction:**

Evaluating the potential effects of non-pharmaceutical interventions on COVID-19 dynamics is challenging and controversially discussed in the literature. The reasons are manifold, and some of them are as follows. First, interventions are strongly correlated, making a specific contribution difficult to disentangle; second, time trends (including SARS-CoV-2 variants, vaccination coverage and seasonality) influence the potential effects; third, interventions influence the different populations and dynamics with a time delay.

**Methods:**

In this article, we apply a distributed lag linear model on COVID-19 data from Germany from January 2020 to June 2022 to study intensity and lag time effects on the number of hospital patients and the number of prevalent intensive care patients diagnosed with polymerase chain reaction tests. We further discuss how the findings depend on the complexity of accounting for the seasonal trends.

**Results and discussion:**

Our findings show that the first reducing effect of non-pharmaceutical interventions on the number of prevalent intensive care patients before vaccination can be expected not before a time lag of 5 days; the main effect is after a time lag of 10–15 days. In general, we denote that the number of hospital and prevalent intensive care patients decrease with an increase in the overall non-pharmaceutical interventions intensity with a time lag of 9 and 10 days. Finally, we emphasize a clear interpretation of the findings noting that a causal conclusion is challenging due to the lack of a suitable experimental study design.

## Introduction

The coronavirus disease 2019 (COVID-19), caused by the severe acute respiratory syndrome coronavirus 2 (SARS-CoV-2) (1), entered the world unexpectedly in 2019, dramatically changing human life (2). Infection occurs through respiratory droplets and contact routes during the incubation period. Outbreaks of the disease first appeared in Wuhan, Hubei Province, China (3), then spread to the United States, Europe, and Asia, reaching all continents. Since the World Health Organization (WHO) declared the disease a pandemic on March 11, 2020 (4), as of June 27, 2022, there have been more than 547 million confirmed cases worldwide and more than 6 million reported deaths (5). However, many confirmed cases required hospitalization for several weeks while others require Intensive Care Unit (ICU) treatment (6). Due to a limited number of hospital beds, mainly ICU beds, many countries have adopted early control measures to prevent viral transmission and to avoid overloading the healthcare system (7, 8). Germany, the largest economic producer in Europe, has also inevitably experienced this pandemic. The first confirmed COVID-19 case in Germany was reported in late January 2020 following contact with an infected colleague from China (9). Afterwards, as of April 17, 2020, the Robert Koch-Institute (RKI) counts over 130,000 confirmed infections and about 4,000 deaths in Germany (10). To anticipate the massive flow of COVID-19, the federal government introduces public closures by closing public spaces such as schools, universities, and restaurants. Additional measures such as the national curfew ban and restrictions on people gatherings were also applied. In principle, people were advised to stay home as long as possible and leave home only for basic needs (11).

Several mathematical and statistical approaches have been developed to investigate the effectiveness of NPIs. Among these studies, Brauner et al. (12) applied a semi-mechanistic hierarchical Bayesian model to estimate the impact of NPIs on the time reproduction numbers in 41 countries during the first wave of the pandemic. They found that closing all educational institutions, limiting gatherings to 10 people or less, and closing face-to-face businesses reduced transmission considerably. The additional effect of stay-at-home orders was comparatively small. Nader et al. (13) used a non-parametric machine learning model to assess the effects of NPIs in relation to how long they have been in place and the effectiveness of NPIs in relation to their implementation date on the daily growth rate (relative increase in cumulative confirmed COVID-19 cases from 1 day to the next). They found that the closure and regulation of schools was the most important NPI, associated with a pronounced effect about 10 days after implementation. Sharma et al. (14) considered a semi-mechanistic hierarchical Bayesian model to examine the effect estimates for individual NPI during Europe's second wave of COVID-19 on daily cases and deaths. They concluded that business closures, educational institution closures, and gathering bans reduced transmission but less than they did during the first wave. Ge et al. (15) used a Bayesian inference model to assess the changing effect of NPIs and vaccination on reducing COVID-19 transmission based on the time reproduction numbers. Their results demonstrate that NPIs were complementary to vaccination in an effort to reduce COVID-19 transmission, and the relaxation of NPIs might depend on vaccination rates, control targets, and vaccine effectiveness concerning extant and emerging variants.

All the studies cited above have shown the effectiveness of NPIs. However, they did not consider the delay effects related to the NPIs implementation. A health effect is frequently associated with protracted exposures of varying intensity sustained in the past (16). The effects of exposing a particular event may not always be limited to the time of its occurrence and may appear with lag times (17). Policy lags are generally understood as unavoidable time delays. While there may exist several possible reasons for a lag, there is no general agreement on its length (18). This can be explained by the high sensitivity of the lagged and baseline exposure terms and also the implication of time-varying confounding variables in the models (19). Similar time lags have been noticed during the COVID-19 outbreak when various non-pharmaceutical interventions (NPIs) were implemented. Different policies may have different levels of effectiveness on disease spread, and the response to these policies is still unclear (20).

The main complexity of modeling and interpreting such phenomena lies in the additional temporal dimension needed to express the association, as the risk depends on both the intensity and timing of past exposures. This type of dependency is defined here as NPIs intensity-lag-response (Hospitalized cases and ICU cases) association. Statistical regression models are used to determine the relationship between predictors and outcomes and then estimate their effects. The Distributed Lag Model (DLM) models the exposure–response relationship and then introduces a series of consequences caused by this exposure to events. In addition, the method is used as well to determine the distribution of the subsequent effects after the occurrence of events (in lag times). This method has been developed for time series data and used in studies of various designs and data structures, cohort, case-control, or longitudinal studies (17). Distributed Lag Models have been successfully applied in epidemiological research (21–24).

Changes in the coronavirus infection dynamic in Germany led to the implementation of a policy like NPIs. The effect of NPIs may not be immediate since NPIs need some time to affect the pandemic situation. Then, it is reasonable to use the time lag concept in the analysis of COVID-19 research. This work aims to study intensity and lag time effects on the numbers of COVID-19 hospital and prevalent intensive care patients diagnosed with polymerase chain reaction tests in Germany from 10 January 2020 to June 2022. In this study, we applied a DLM to the number of COVID-19 hospital patients (Hospitalized cases) in Germany and the number of COVID-19 prevalent intensive care patients (ICU cases), considering all non-pharmaceutical interventions implemented in Germany and estimated their delayed effects. The results provide policymakers with essential information to make more informed decisions, considering the effect of NPIs, and their lag time in managing possible future pandemics.

## Methods

### Data description

Real data about the number of hospital patients (Hospitalized cases), prevalent intensive care patients (ICU cases), overall non-pharmaceutical intervention intensity (*NPIs*) for each of the 16 German states, the proportion of people who received at least two doses (*V*2) in Germany from January 2020 to June 2022, were extracted from the corona Daten platform site (https://www.corona-datenplattform.de). The first two variables (i.e., Hospitalized and ICU cases) were summed over states to obtain German countrywide data for our analysis.

The overall intensity of non-pharmaceutical interventions for Germany was computed using


(1) $$\begin{array}{l} NPIs_{t}=\overset{16}{\underset{f=1}{\sum }}{ℑ}_{f}NPI_{f,t} \end{array}$$

where ℑ_*f*_ is state *f* relative population share in Germany, and *NPI*_*f, t*_ is the intensity of all NPIs implemented in the state *f* at time *t*. The whole German data was split into two sets to obtain a dataset before vaccination (from January 2020 to December 2020 data), a dataset with vaccination (from December 2020 to June 2022 data), and the entire dataset (from January 2020 to June 2022).

### Distributed lag generalized linear model based regression

To assess the lag time effects of non-pharmaceutical interventions on COVID-19 dynamics, we used a DLM on each of the three datasets, considering the daily number of hospital patients (Hospitalized cases) and the number of prevalent intensive care patients (ICU cases) as response variables and overall NPIs intensity as exposure (predictor). Only time (variable date) is considered as a confounding variable for the data before vaccination. In addition to the time, the proportion of people who received at least two doses (*V*2) was considered a confounding variable for the data with vaccination and the entire data. The analyses were conducted within the statistical environment R version 4.0.3 (25) using the package **dlnm** (26).

Mathematically, a general model for time series data of outcomes *y*_*t*_ at time *t* can be written as:


(2) $$\begin{array}{l} g\left(\mu_{t}\right)=\alpha +\overset{J}{\underset{j=1}{\sum }}s_{j}\left(x_{tj};\beta_{j}\right)+\overset{K}{\underset{k=1}{\sum }}\phi_{k}u_{tk}, \end{array}$$

where μ_*t*_ = E(*y*_*t*_) is the expected response for the day *t*, *g* is a monotonic link function (here *g* = *log*), and *y*_*t*_ (*t* = 1, …*n*) arises from a time series which is assumed to have an exponential family distribution (27). The function *s*_*j*_ is a smoother of the relationships between the variables *x*_*j*_ and the linear predictor, expressed by the parameter **β*_j_***. Lastly, the *u*_*k*_ variables include other predictors with effects specified by the related coefficients ϕ_*k*_. In this paper, we considered a set of variables *x*, which is overall NPIs intensity (*NPIs*) and two sets of variables *u*_1_ and *u*_2_, which are, respectively, date (*t*) and the proportion of people who received at least two doses (*V*2). We did a transformation to use nonlinear influences of the variable date (*t*) and captured its effect changing over time. This transformation is described in matrix notation as


$$\begin{array}{l} f\left(t,\alpha \right)=z_{t}\alpha \end{array}$$

where *z*_*t*_ is the *t*^*th*^ row of the matrix ***Z***. The transformation on a variable date (time) consists of using *ns*(*t, df*), where *df* corresponds to the degree of freedom and *ns*, the natural spline function. The parameters for the natural spline are implicitly captured in the *ns* function of the R package *splines*. The matrix ***Z*** is generated automatically, and the parameters for the natural spline are implicitly captured by the function *ns*.

For the models considered, we assumed the influence of *NPIs* and the proportion of people who received at least two doses (*V*2) to be linear, with no basis transformation. We assumed this since, from a preliminary investigation based on the Akaike Information Criterion (AIC), the linear model outperforms the non-linear model. The general notation for exposure-response linear relationships could be


(3) $$\begin{array}{l} \begin{array}{cc} s\left(x_{t};\beta \right) & =\overset{L}{\underset{\ell =0}{\sum }}\beta_{\ell }x_{t-\ell } \end{array} \end{array}$$

where ℓ∈[0, *L*] is the lag duration, *L* (here *L* = 30) is the lag period over which exposure to *x* is assumed to affect the count change at time *t*, *x*_*t*−ℓ_ is exposure intensity at time *t*−ℓ, and β_ℓ_ is the contribution from a unit increase in exposure *x* occurring at time *t*−ℓ in the past to a given count change measured at time *t*. For a more detailed description of the general theory on time-lagged models, we refer the reader to Gasparrini (26). The models are specified as indicated in Table 1 for the three datasets, along with the maximum number of degrees of freedom (*df* max) considered.

**Table 1:** Summary of model features. *df* max is the maximum degree of freedom considered.

**Dataset**	**Model**	***df* max**
Data before vaccination	$g\left(\mu_{t}\right)=\alpha +ns\left(t,df\right)+\overset{L}{\underset{\ell =0}{\sum }}\beta_{\ell }NPIs_{t-\ell }$	10
Data with vaccination	$g\left(\mu_{t}\right)=\alpha +ns\left(t,df\right)+\overset{L}{\underset{\ell =0}{\sum }}\beta_{1\ell }NPIs_{t-\ell }+\overset{L}{\underset{\ell =0}{\sum }}\beta_{2\ell }V2_{t-\ell }$	15
Entire data		25

To implement delayed effects, the variables *NPIs*, *V*2, and date (*t*) are used to predict the two response variables (Hospitalized cases, ICU cases). The analysis is based on the models in Table 1, fitted through a generalized linear model with the Quasi-Poisson family, with natural splines of time with different degrees of freedom (*df* = 1 to *df* max) to describe long-time trends. A comparison was made between models with varying numbers of degrees of freedom using modified Akaike information criterion for models with overdispersed responses fitted through quasi-likelihood (28), given by:


(4) $$\begin{array}{c} QAIC=-2L\left(\hat{\theta }\right)+2\hat{\phi }k, \end{array}$$

where *k* is the number of parameters, whereas *L* is the log-likelihood of the fitted model with parameters $\hat{\theta }$ and $\hat{\phi }$, the estimated overdispersion parameter. Minimizing this criterion has led to the best model.

Sensitivity analyses were conducted to assess the impact of choices regarding the number of degrees of freedom (*df*) of the models. Specifically, we examine changes in the estimated overall effect associated with varying *df* for specifying the date or seasonal trend.

## Results

Results for simple DLMs, assuming a linear relationship between response variables (number of hospital patients and number of prevalent intensive care patients) and all non-pharmaceutical interventions implemented in Germany (data before vaccination), and the proportion of people who received at least two doses (data with vaccination and entire Germany data) with a maximum lag equal to 30 days are summarized as follows. The Quasi AIC values for the number of degrees of freedom, *df* = 1 to *df* max (Table 1), are shown in Supplementary Figure S6. When used to compare different modeling choices with varying numbers of degrees of freedom, the Quasi AIC led to a parsimonious model, with 7 *df* for the data before vaccination (Supplementary Figures S7A, D), 15 *df* for the data with vaccination (Supplementary Figures S7B, E), 23 *df* for the whole German COVID-19 Hospitalized cases (Supplementary Figure S7C), and 19 *df* for whole German COVID-19 ICU cases (Supplementary Figure S7F) to describe the overall effect of exposures-lag on response variables.

**Figure 1:**
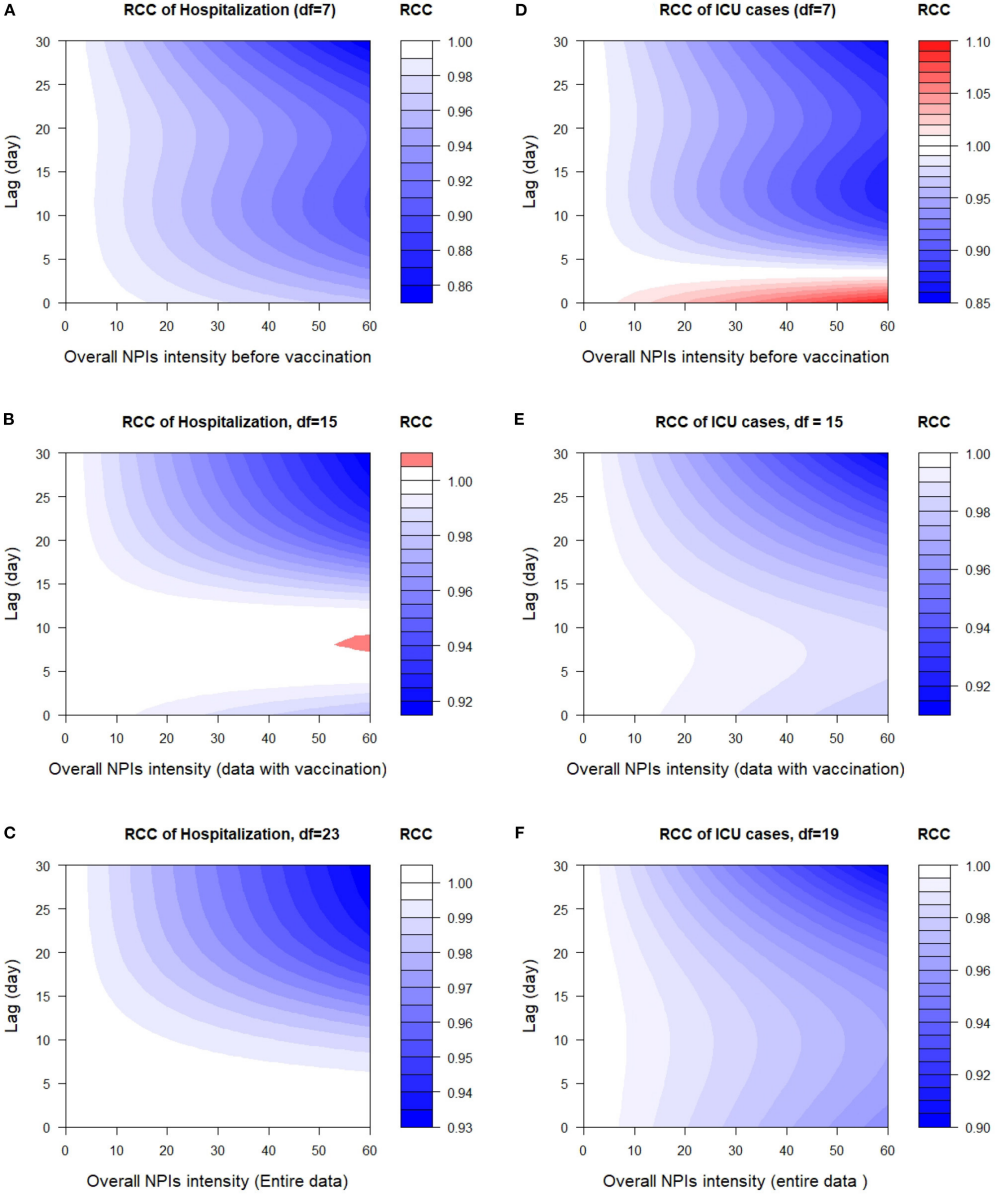
2-D plot of relative count change (RCC) along NPIs and lags on the number of hospital patients (Hospitalized cases) and the number of prevalent intensive care patients (ICU cases). **(A)** RCC of Hospitalization (df = 7), **(B)** RCC of Hospitalization, df = 15, **(C)** RCC of Hospitalization, df = 23, **(D)** RCC of ICU cases (df = 7), **(E)** RCC of ICU cases, df = 15, and **(F)** RCC of ICU cases, df = 19.

An overall graph of the effect of NPIs on the number of hospital patients (Hospitalized cases) and the number of prevalent intensive care patients (ICU cases) is provided in Figure 1, showing heat maps of the relative count change (RCC) of response variable along overall NPIs intensity and lags. Overall, the figure indicates that NPIs have an effect on the response variables before the vaccination program (Figures 1A, D) than on the response variables in the other two datasets (data with vaccination and the entire Germany data). The effect of NPIs is somewhat more immediate on Hospitalized cases before vaccination than on ICU cases. From 40% to 60% overall NPIs intensity, the mean relative count change of hospitalization before vaccination decreased from 1 to 0.85. In addition, a delay of 5 days was observed in the effect of overall NPIs on the ICU cases before vaccination, with a relative count change of 0.85 from 45% overall NPIs intensity.

Concerning data with vaccination and the entire German data, the lag time effects of non-pharmaceutical interventions on the number of hospital patients (Hospitalized cases) are immediate. However, the relative count change in hospitalization (data with vaccination) is high between lags 9 and 10 days from 55% to 60% overall NPIs intensity in Germany (see Figure 1B). The maximum effect of all non-pharmaceutical interventions implemented in Germany on Hospitalized and ICU cases during the vaccination programme is reached approximately at lag 25–30 days at 45–60% overall NPIs intensity.

**Figure 2:**
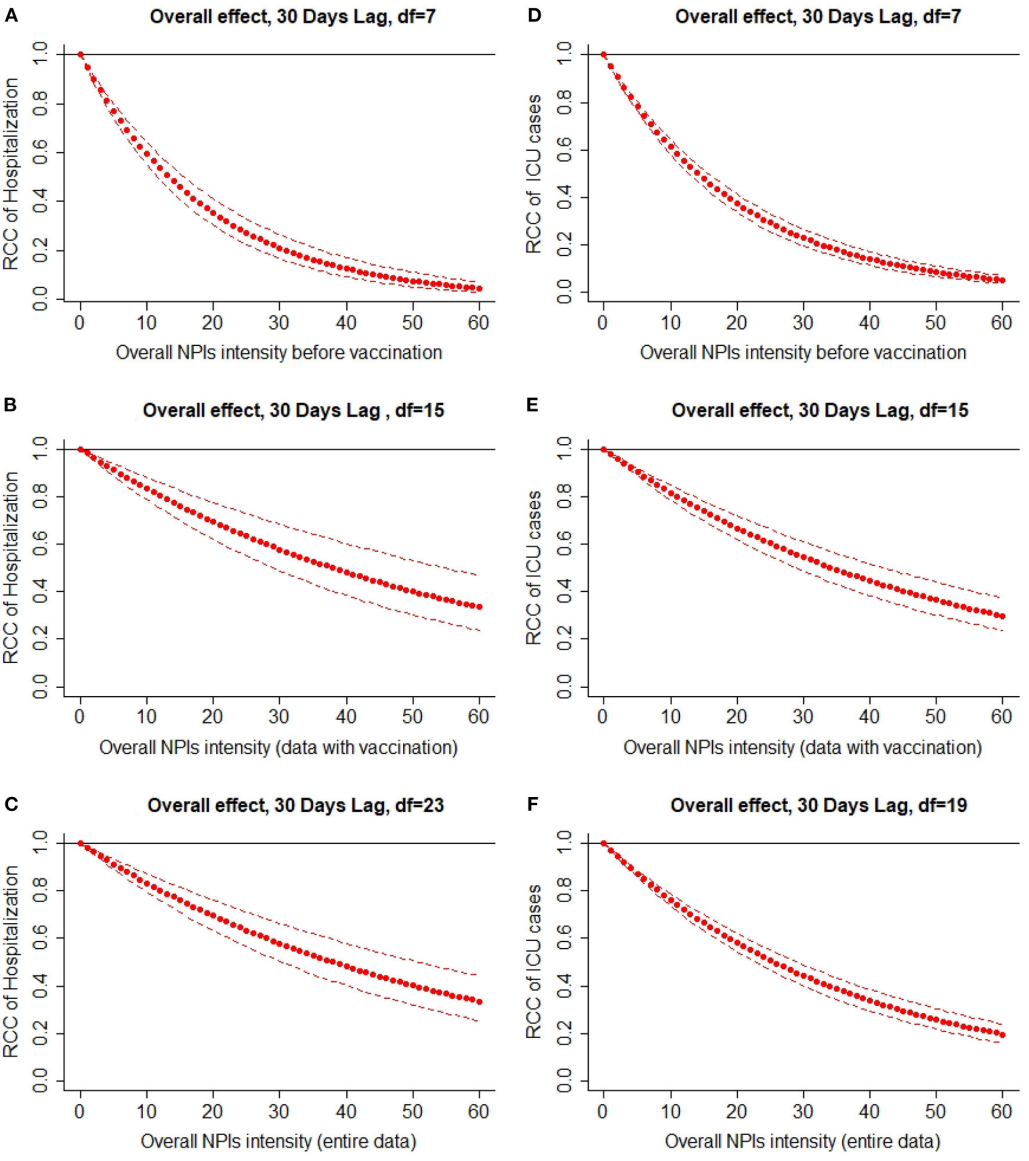
Overall lag effect of NPIs intensity on the number of incident hospital patients (Hospitalized cases) and the number of prevalent intensive care patients (ICU cases) with 95% confidence intervals. **(A)** Overall effect, 30 days lag, df = 7, **(B)** overall effect, 30 days lag, df = 15, **(C)** overall effect, 30 days lag, df = 23, **(D)** overall effect, 30 days lag, df = 7, **(E)** overall effect, 30 days lag, df = 15 and **(F)** overall effect, 30 days lag, df = 19.

Figure 2 presents the results from the sensitivity analyses, showing the overall effect of all NPIs implemented in Germany, summing up the contributions for the 30 days of lag considered in the analysis. There was an overall decrease in the number of patients in hospital and intensive care units with increasing overall NPIs intensity. This relative count change (RCC) was canceled out for the data before vaccination and reached its minimum value of around 0.3 for the data with vaccination and the entire data at 55–60% overall NPIs intensity.

## Discussions

This study used a Distributed Lag Linear Model (DLM) to evaluate the lag time effects of non-pharmaceutical interventions on the number of COVID-19 hospital patients and the number of prevalent COVID-19 intensive care patients. Based on the results of this analysis, it is important to investigate both the lag and magnitude of NPIs impact jointly (17).

An epidemiological discussion of DLM choice emphasized the need to balance capturing detail and allow for interpretation (23). Despite its conceptual simplicity, DLM specifications allow for a wide range of models, including simple previously used models and more complex variants. There is a challenge in choosing between alternatives when there is an abundance of choices (number of degrees of freedom, maximum lag). We used quasi-Akaike information criteria to guide the selection of the number of degrees of freedom (*df*) of the variable *time*. Due to the lack of consensus about what constitutes an optimal model, sensitivity analyses are particularly important for assessing how key conclusions depend on the model's number of degrees of freedom.

The results of our study revealed that NPIs have a positive effect on the number of hospital patients (Hospitalized cases) and of prevalent intensive care patients (ICU cases) for all the datasets (data before vaccination, data with vaccination or the entire COVID-19 German data) since the overall non-pharmaceutical intervention decrease the number of incident hospital patients (Hospitalized cases) and the number of prevalent intensive care patients (ICU cases). These results are similar to a previous study which showed that some interventions are effective in reducing the advent of the pandemic (29). We found that the first reducing effect of NPIs on the number of prevalent intensive care patients before vaccination cannot be expected before a time lag of 5 days. As 5 days seems to be a short delay effect, it might be possible that already the announcement of planned NPI introductions from policy makers have an impact on the social behavior such as contact activities and hence on the pandemic dynamics. However, our results also suggest that the main effect is after a time lag of 10–15 days.

However, the results contrast with a previous study which evaluated NPIs effects on the COVID-19 pandemic in Germany and three other European countries using the Granger Causality test (30). In addition, previous studies have focussed on the effect of NPIs on infectious cases and death (11, 14), recovered cases (11), daily growth rate (13), or time-varying reproduction number (12, 15) contrary to this study. The contradiction could be due to the fact that the previous study took into account the effect of NPIs on the number of infections in the second wave, whereas we evaluated the way how NPIs are associated with a decrease in the number of patients in hospitals and intensive care units diagnosed with PCR in several waves. We note that the number of infections in the general population usually depends on several non-infection-related factors, such as testing behavior and the day of the week and is therefore often subject to reporting bias and delays. Thus, this outcome is less specific and prone to higher statistical noise than the number of patients in hospital and intensive care units. In addition to the DLM analyses, the Granger causality test methodology has been applied to our data set. They resulted in a similar conclusion, even though all-time series have to be transformed by the second differences to reach stationarity and decomposed with respect to time trends and seasonality (data not shown).

The advantage of applying DLM is that it is flexible and provides a comprehensive scheme for interpreting outcomes from exposure-lag-response associations contrary to other statistical approaches (16). The main limitation of our analysis is that our results cannot—strictly speaking—be interpreted as causal effects; they are associations. To increase the ability to infer causality, pragmatic study designs such as the SMART (Sequential, Multiple-Assignment Randomized Trial), stepped wedge, and preference designs could have been an option (31). An interesting design and analysis is also the trial emulation approach (32), where cluster-randomized trials are mimicked. However, in Germany, the introduction, timing, and intensity of NPIs were quite homogeneously distributed across Germany (see Supplementary material); hence, the above-mentioned designs were hardly feasible in practice.

Obtaining “zero Hospitalized or ICU COVID-19 cases” may not be achievable, but reducing the number to a level that can be managed by the health system might be a feasible goal. This paper considered a bundle or the overall intensity of NPIs implemented in Germany; an isolation of specific NPIs is hardly feasible due to high correlations. However, a society's ability to fight a pandemic can be influenced by various factors, including how well the public health care system works, how the government manages risk, transparency of information flow, how it is driven by politics, corporate and citizen compliance, etc. Consequently, further studies are needed to investigate what determines whether or not the COVID-19 pandemic is controlled.

An application to the COVID-19 data from Germany indicates that the Distributed Lag Model can be used to assess the effect of control measures dictated by health policies with changes in the transmission dynamics of the studied disease. In addition, using them can assist policymakers in planning appropriate and timely strategies and allocating resources (20). Our results can inform political decision makers regarding how long NPIs need to be implemented to take effect on controlling the COVID-19 dynamics in hospitals. However, we focused only on this aspect. Beyond that, NPIs create tremendous economic and social collateral damages of multifaceted dimensions such as psychological long-term effects on mental health of children due to long school closures and contact distancing. Thus, political decision makers need to trade off NPIs effects on hospitals against the collateral damages of NPIs in the society.

## Data availability statement

The datasets presented in this study can be found in online repositories. The names of the repository/repositories and accession number(s) can be found below: https://www.corona-datenplattform.de.

## Author contributions

YM, RGK, and MW contributed to the study conception and design. YM carried out data analysis and wrote the initial manuscript draft. MW contributed to data collection, supervised data analysis, and revised the manuscript. RGK supervised data analysis and revised the manuscript. All authors read and revised the initial manuscript. All authors contributed to the article and approved the submitted version.
