# Supplementary material for: Intensity and lag-time of non-pharmaceutical interventions on COVID-19 dynamics in German hospitals
Source: Front Public Health. 2023 Mar 6;11:1087580. doi: 10.3389/fpubh.2023.1087580 (PMC10025539; doi:10.3389/fpubh.2023.1087580)
Supplement: Supplementary file 1 [file Data_Sheet_1.PDF]

Supplementary Material

1 SUPPLEMENTARY FIGURES

1.1 Figures

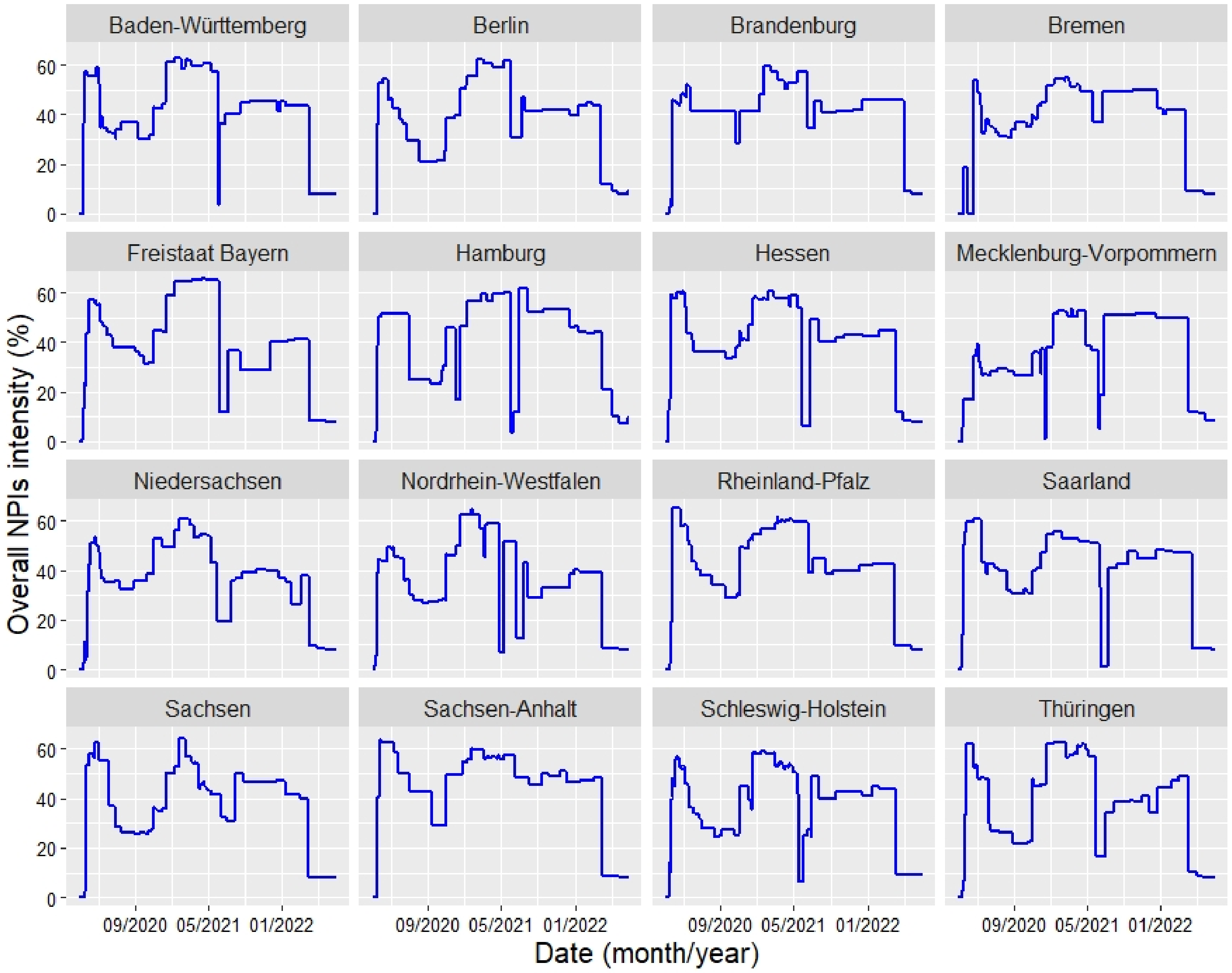

Figure S1. Daily overall non-pharmaceutical intervention intensity per Germany federal state.

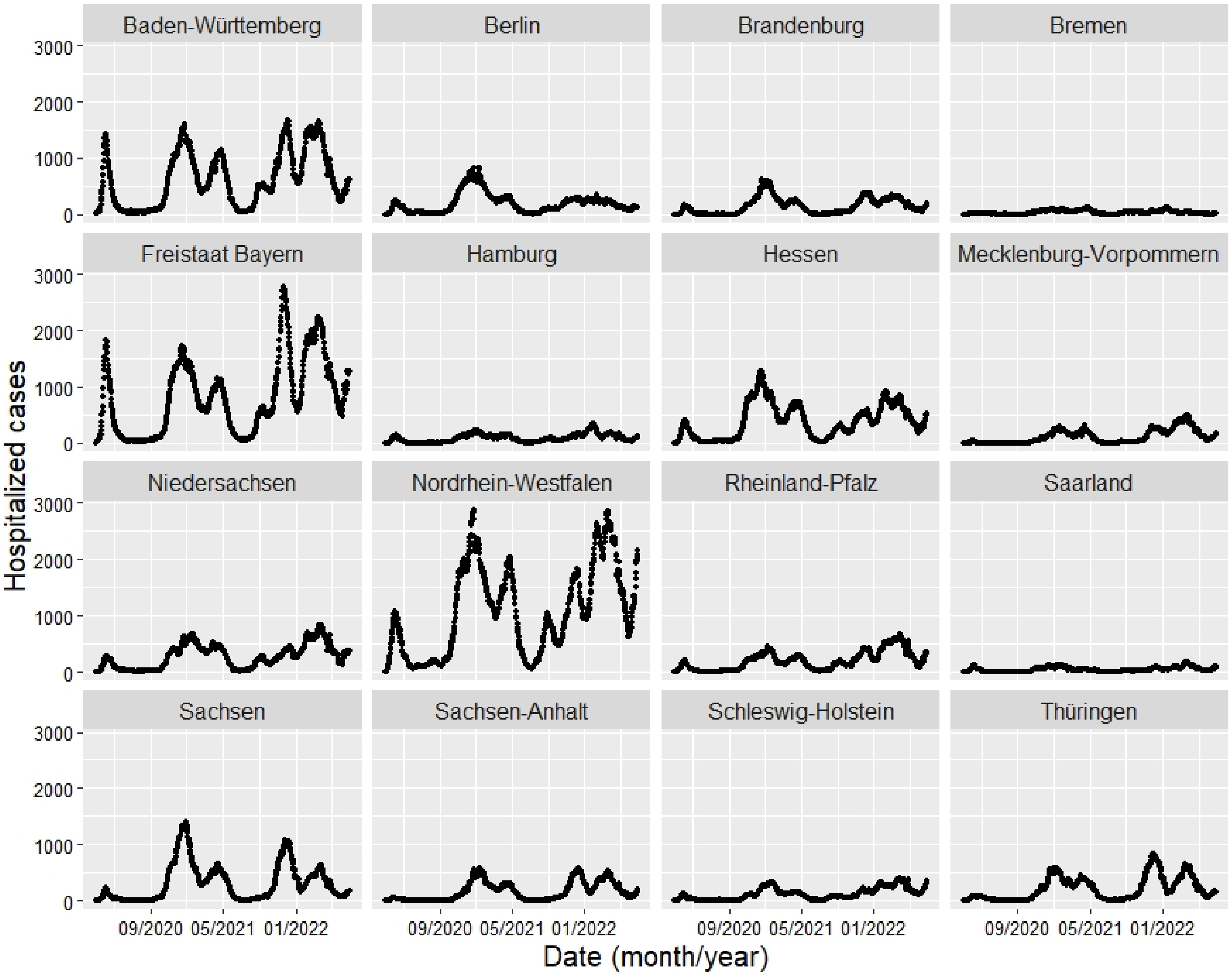

Figure S2. Daily number of COVID-19 incident hospital patients (Hospitalized cases) per Germany federal state.

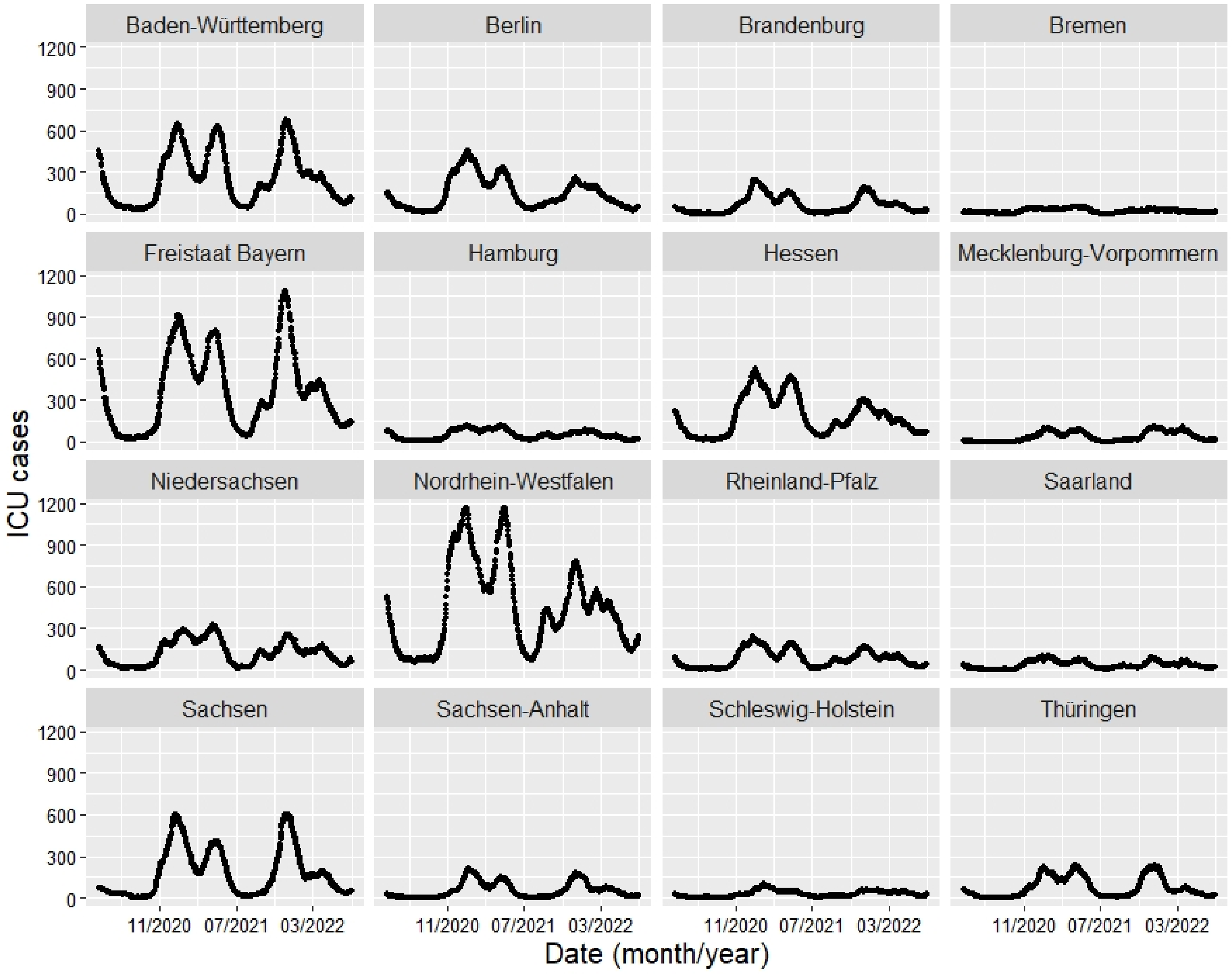

Figure S3. Daily number of COVID-19 prevalent intensive care patients (ICU cases) per Germany federal state.

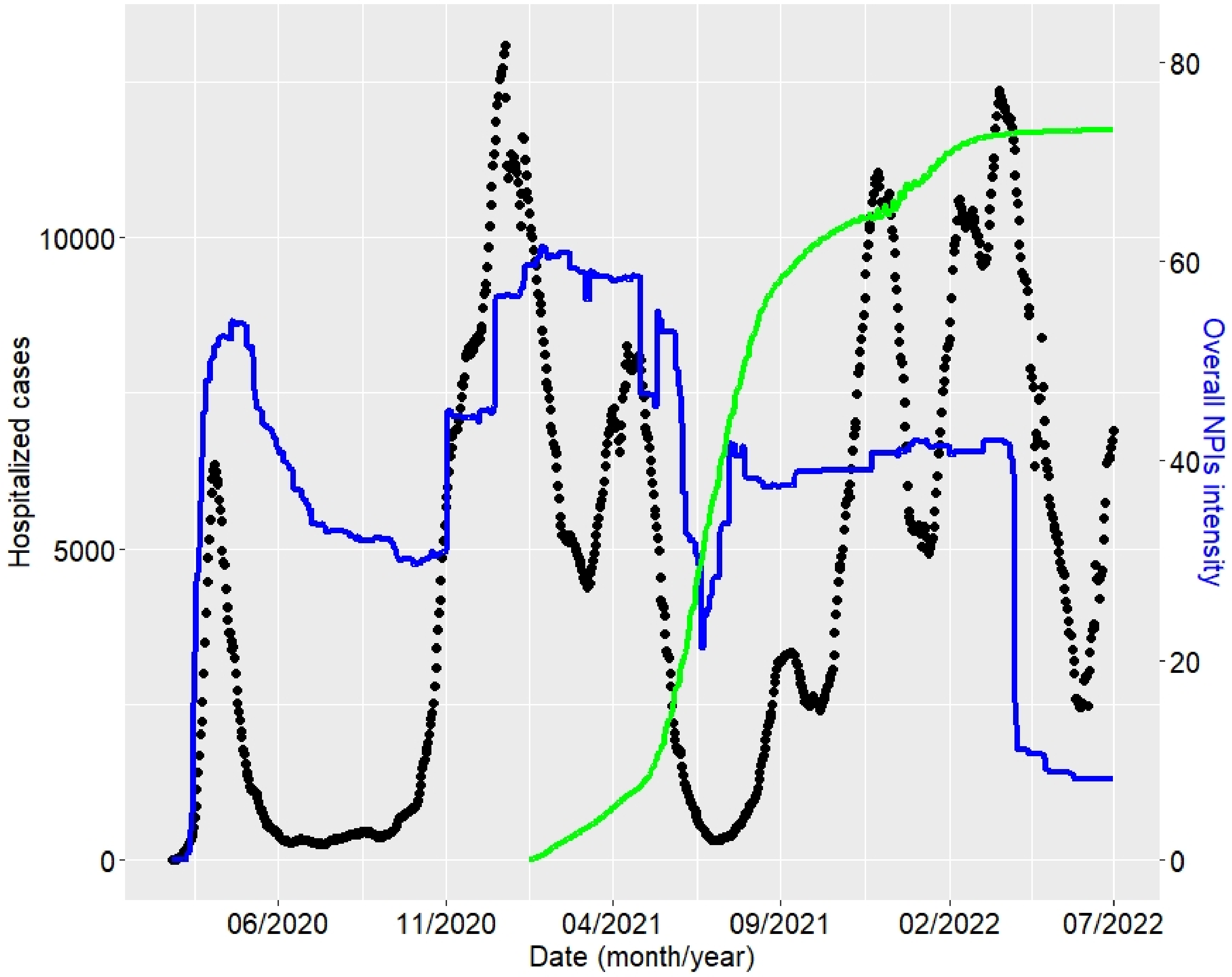

Figure S4. Whole German daily number of COVID-19 incident hospital patients (Hospitalized cases). The blue and green lines have the same scale of graduation and are respectively daily overall NPIs intensity and $V2$ (proportion of people who received at least two doses).

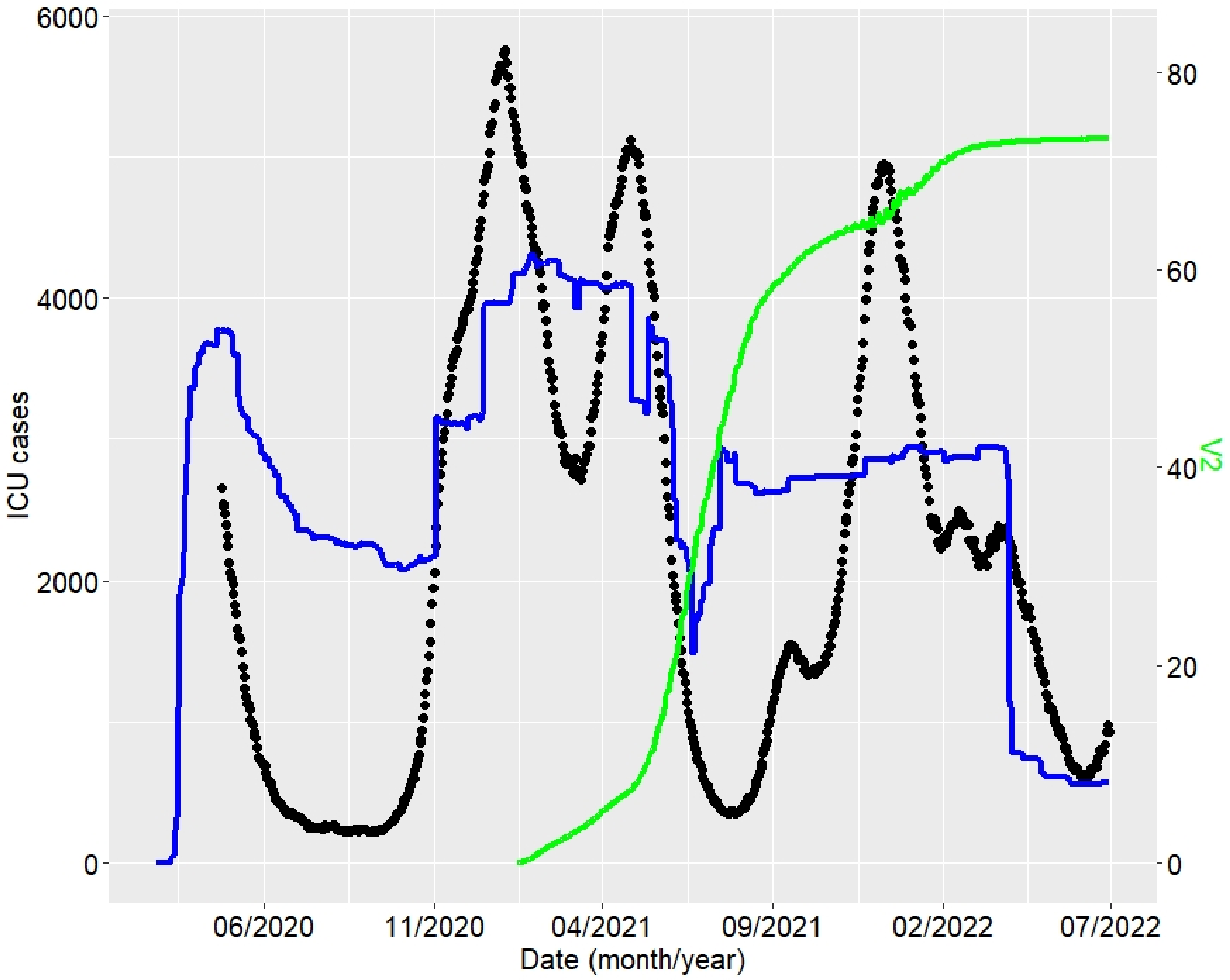

Figure S5. Whole German daily number of COVID-19 prevalent intensive care patients (ICU cases). The blue and green lines have the same scale of graduation and are respectively daily overall NPIs intensity and $V2$ (the proportion of people who received at least two doses).

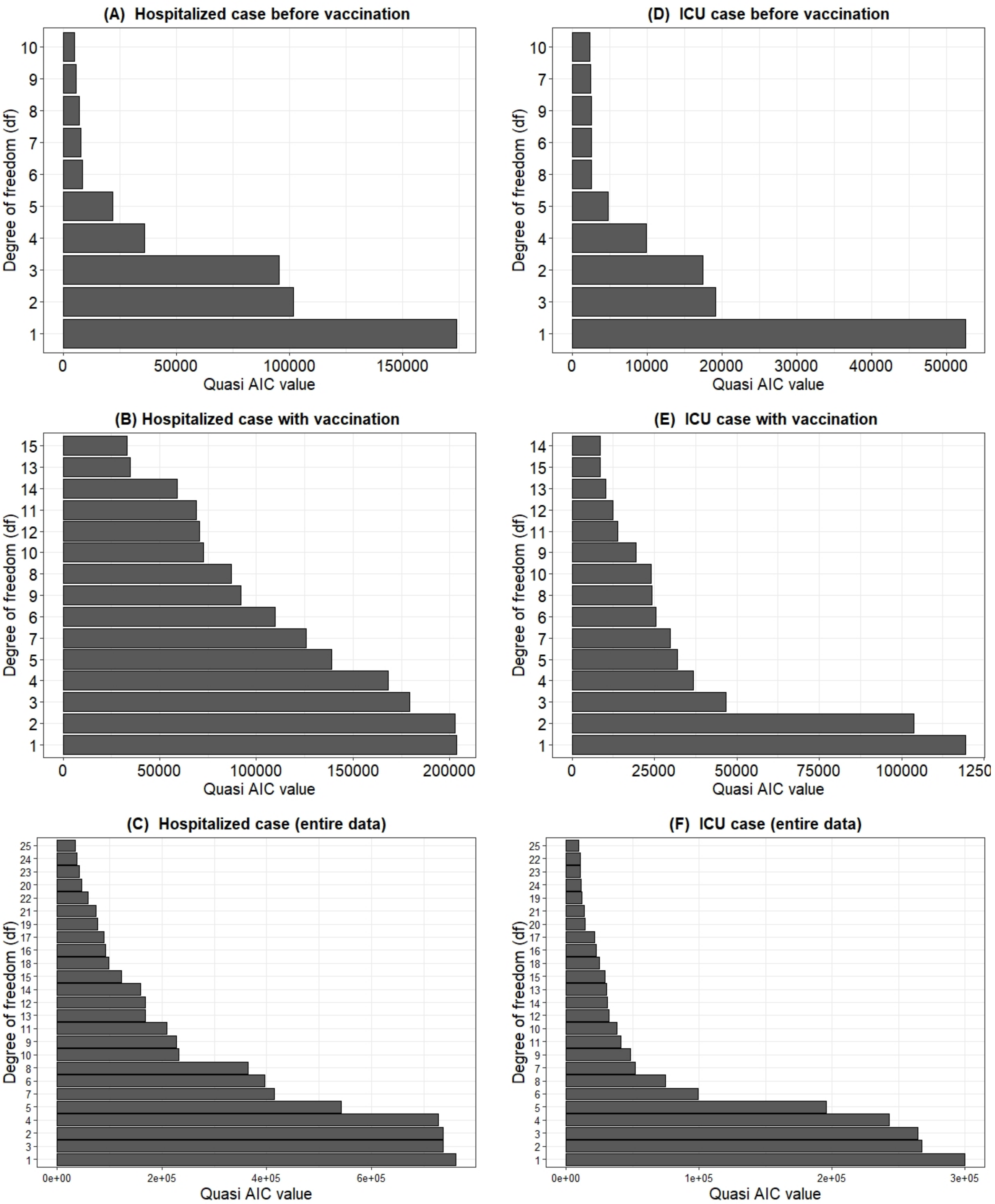

Figure S6. Quasi AIC value of various degrees of freedom (df).

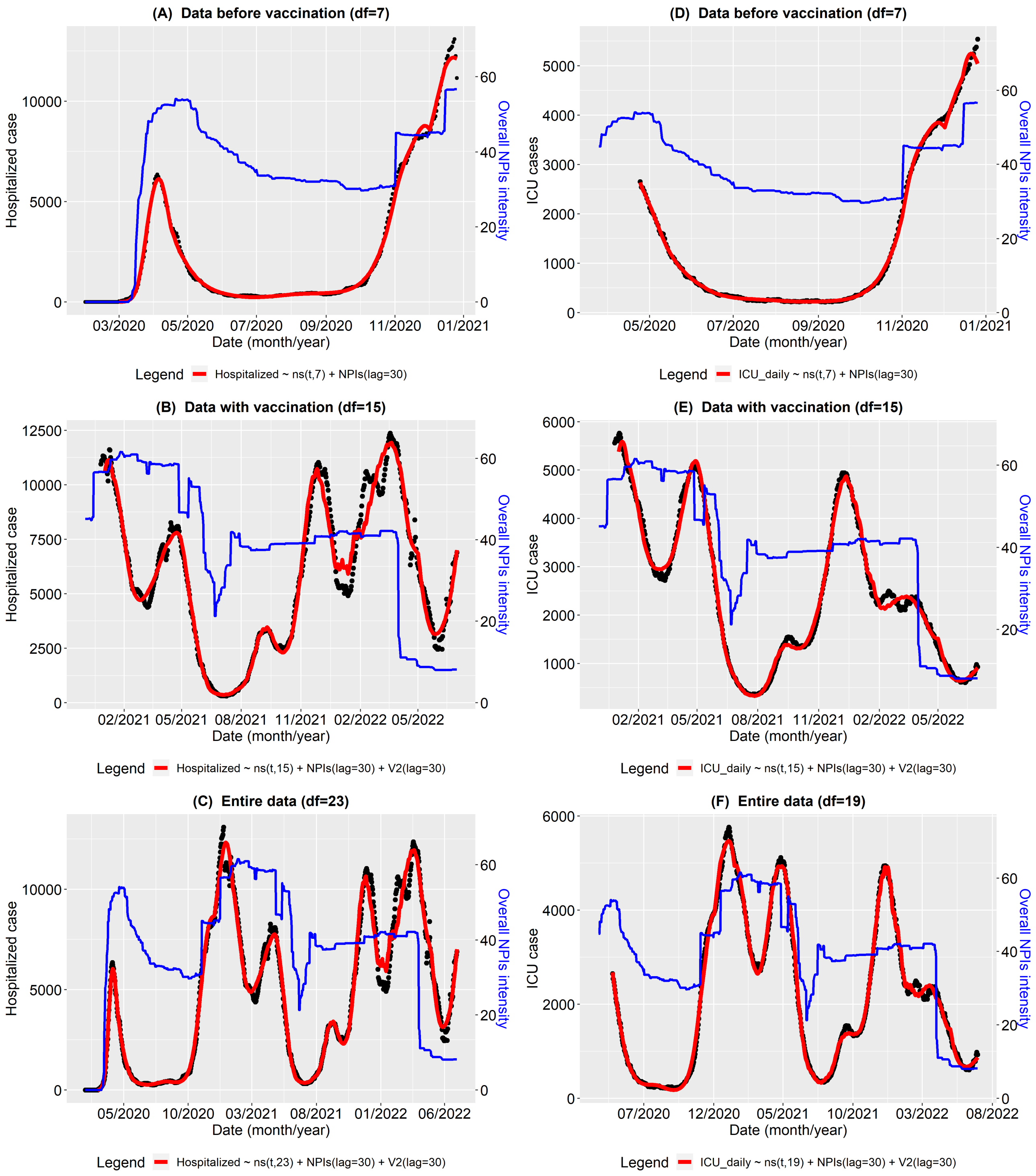

Figure S7. Distributed lag linear models fitting on the number of COVID-19 incident hospital patients (Hospitalized cases) and the number of COVID-19 prevalent intensive care patients (ICU cases).
